# Progress of Epidemics in Thirty-Six Towns and Districts

**Published:** 1856-10

**Authors:** 


### PROGRESS OF EPIDEMICS.

### LOCAL REPORTS OF EPIDEMIC AND ENDEMIC DISEASES

*During the Months of June, July, and August,* 1856.

| Place. | County. | Lat. | Long. | Observer. |
|---|---|---|---|---|
| St.Mary, Scilly | Cornwall - | 49.50 N. | 6.18 W. | J. G. Moyle, Esq. |
| Teignmouth - | Devonshire - | 50.32 N. | 3.29 W. | W. C. Lake, Esq. |
| Canterbury - | Kent - - | 51.17 N. | 1. 4 E. | G. Rigden, Esq.<br>James Reid, Esq. |
| Chatham - | Kent - - | 51.24 N. | 0.14 E. | F. J. Brown, M.D. |
| Wandsworth - | Surrey - - | 51.28 N. | 0. 7 W. | G. E. Nicholas, Esq. |
| Putney - - | Surrey - - | 51.28 N. | 0. 8 W. | R. H. Whiteman, Esq. |
| Up. Holloway | Middlesex - | 51.32 N. | 0.03 E. | W. B. Kesteven, Esq. |
| Wanstead - | Essex - - | 51.32 N. | 0. 2 W. | F. Collins, M.D. |
| Swansea - | Glamorgansh. | 51.38 N. | 3.50 W. | W. H. Michael, Esq. |
| Saffron Walden | Essex - - | 52. 3 N. | 0.12 E. | H. Stear, Esq. |
| Bedford - - | Bedfordshire - | 52. 8 N. | 0.51 W. | T. H. Barker, M.D. |
| Sharnbrook - | Bedfordshire - | 52.12 N. | 0.40 W. | R. S. Stedman, Esq. |
| Newpt.Pagnell | Buckinghams. | 52.10 N. | 0.42 W. | G. O. Rogers, Esq. |
| Wellingbro' - | Northamptons. | 52.20 N. | 0.40 W. | B. Dulley, Esq. |
| Beccles - - | Suffolk - - | 52.25 N. | 1.48 E. | W. E. Crowfoot, Esq. |
| Thetford - | Norfolk - - | 52.26 N. | 0.45 E. | H. W. Bailey, Esq. |
| Dudley - - | Staffordshire - | 52.30 N. | 2.10 W. | J. H. Houghton, Esq. |
| Barrowden - | Rutland - | 52.34 N. | 0.38 W. | H. J. Swann, Esq. |
| Wisbeach - | Cambridgesh. | 52.39 N. | 0. 5 E. | W. H. Hole, Esq. |
| East Dereham | Norfolk - - | 52.40 N. | 0.57 E. | J. Vincent, M.D. |
| Pontesbury - | Shropshire - | 52.43 N. | 2.50 W. | Wm. Eddowes, Esq. |
| Nottingham - | Nottinghamsh. | 52.50 N. | 1.10 W. | T. Robertson, M.D. |
| Burton-on-Trt. | Staffordshire - | 52.53 N. | 1.53 W. | S. Thomson, M.D. |
| Wrexham - | Denbighshire - | 53. 2 N. | 3. 1 W. | E. Williams, M.D. |
| Hawarden - | Flintshire - | 53.11 N. | 3. 2 W. | T. Moffat, M.D. |
| Lincoln - | Lincolnshire - | 53.12 N. | 0. 5 W. | S. Lowe, Esq. |
| Alford - - | Lincolnshire - | 53.15 N. | 0. 6 E. | R. U. West, M.D. |
| Staveley - | Derbyshire - | 53.15 N. | 1.20 W. | G. B. Thorpe, Esq. |
| Gainsborough | Lincolnshire - | 53.23 N. | 0.47 W. | D. Mackinder, M.D. |
| Liverpool - | Lancashire - | 53.24 N. | 2.59 W. | Thos. Bickerton, Esq. |
| Warrington - | Lancashire - | 53.25 N. | 2.32 W. | C. N. Spinks, Esq. |
| Wigan - - | Lancashire - | 53.32 N. | 2.33 W. | W. I. Cox, Esq. |
| Bolton - - | Lancashire - | 53.35 N. | 2.19 W. | W.H. Pendlebury, Esq. |
| York - - | Yorkshire - | 53.58 N. | 1. 3 W. | W. Procter, Esq. |
| Wst. Auckland | Durham - | 54.45 N. | 1.40 W. | G. Todd, Esq. |
| Rothbury - | Northumberld. | 55.25 N. | 1.50 W. | E. C. Summers, Esq. |

### QUARTERLY STATEMENT—No. VII.

[*The dates denote that the disease appeared in the weeks then ending.*]

SCARLET FEVER.

Canterbury..June 6-20, Aug. 22
Chatham..June 6, 13, 27, all July, Aug.
Wandsworth..July 4 8-22
Upper Holloway..August 29
Swansea..June 20, July 4, 18, 25, Aug.
Saffron Walden..Every week [8, 22, 29
Sharnbrook..All June, July 18, Aug.
Newport Pagnell..June 6-13 [1-15
Wellingborough..All June, July 4-18,
Beccles..June 6 [Aug. 29
Thetford..June 13, 27, July 4, 11, 25,
Dudley..All July [Aug. 15-29
East Dereham..June 20, all July & Aug.

Wisbeach..All July
Pontesbury..July 11-25, Aug. 1-8
Nottingham..July 4, 25
Wrexham..June 6-20
Gainsborough..August 8
Liverpool..Every week [1-15
Warrington..June 13-27, July 25, Aug.
Bolton-le-Moors..All June, July 4, 18, August 8, 29

MEASLES.

Canterbury..June 6-13, July 25
Chatham..June 13, July 4-11, Aug. 15
Wandsworth..All July, Aug. 1, 8, 22
Putney..August 8-15
Upper Holloway..June 6-13
Swansea..July 18, August 8
Bedford..July 18-25, all August
Sharnbrook..All June and July
Newport Pagnell..June 6, July 18-25, August 1
Wellingborough..All June and July
Thetford..June 6, 13, 27, July 4, 18, 25, August 8, 22
Barrowden..July 11-25, all August
Wisbeach..July 11-18, August 22-29
Pontesbury..July 25, August 1, 8, 29
Hawarden..July 4
Gainsborough..August 8-15
Liverpool..Every week
Wigan..All June [25, Aug. 8
Bolton-le-Moors..All June, July 4, 11,

SMALL-POX.

Canterbury..June 6-13, July 18-25,
Chatham..June 6-13, Aug. 22 [Aug. 8
Wandsworth..All June and July, Aug.
Bedford..July 11 [1, 8
Wellingborough..Every week
Dudley..July 25, August 8-29
Pontesbury..July 4, 11
Liverpool..Every week
York..June 6, 13, 27, July 18-25, Aug. 1
West Auckland..All June

HOOPING COUGH.

Teignmouth..All June, July 4, 11, 25
Canterbury..Every week
Chatham..June 6
Wandsworth..June 27, July 4, Aug. 1-8
Upper Holloway..June 6
Saffron Walden..Every week
Bedford..August 15-22
Thetford..All June and July, Aug. 15,
Wisbeach..All June, July 4 [29
Pontesbury..July 18-25, Aug. 1-8
Burton-on-Trent..July 11, 25
Alford..Every week
Liverpool..Every week
Warrington..August 15-29
Wigan..June 13-27, all July, Aug. 1
Bolton-le-Moors..June 13-20, July 11-25, August 15
York..June 13-27, July 4, 11, 25, Aug. 8
Rothbury..June 13-27

CROUP.

Canterbury..June 27, July 4, Aug. 29
Swansea..June 13, 27, July 11, 25, August 1, 29
Wellingborough..June 6, August 29
Beccles..June 6-20, July 18-25
Barrowden..June 1
Burton-on-Trent..July 11
Wrexham..August 22
Liverpool..June 27, July 11, Aug. 1 22
Wigan..All June
York..June 27, August 29

CATARRH.

Scilly Islands..June 6-20 [Aug. 8-29
Teignmouth..June 13-27, July 11-18,
Chatham..All June, July 11, Aug. 1-15
Wandsworth..August 1, 29
Putney..June 13-20, July 18
Upper Holloway..June 6-13, Aug. 8
Wanstead..June 6-13
Sharnbrook..August 15-29
Newport Pagnell..August 22 [1, 15
Beccles..June 20-27, July 4, 18, Aug.
Thetford..June 6, 13, 27, July 11, 25,
Barrowden..July 11 [Aug. 8 15
Wisbeach..Every week except Aug. 1
Burton-on-Trent..July 4
Gainsborough..All June
Liverpool..Every week
Bolton-le-Moors..July 18-25, Aug. 1-8
Rothbury..July 4-18

INFLUENZA.

Scilly Islands..All July
Teignmouth..June 13-20, July 4-11
Chatham..August 15
Wandsworth..June 6
Upper Holloway..August 8-15
Wanstead..June 27, July 4
Swansea..August 1, 15, 22
Newport Pagnell..All June, July 4, 18, 25, August 1, 15
Wellingborough..June 6-13
Beccles..All June
Thetford..All June, July 4-11, Aug. 8,
Wrexham..June 20-27 [22, 29
Alford..June 20-27
Bolton-le-Moors..July 11, 25, Aug. 1, 15

ERYSIPELAS.

Scilly Islands..August 8, 22
Teignmouth..August 22
Canterbury..June 6-13, Aug. 8-22
Chatham..June 6-20, July 4, 18, 25, August 22-29
Wandsworth..June 13, July 4-11, Aug.
Putney..August 8-22 [15
Upper Holloway..August 29
Wanstead..July 4, 11, 25, Aug. 1
Swansea..June 13-27

Saffron Walden..June 6, 20, 27
Bedford..August 1, 22
Sharnbrook..July 4, August 15
Newport Pagnell..June 6, 20, 27, July 4-11, August 1, 29
Wellingborough..July 25, all August
Thetford..June 13, 27, July 11-25, August 1, 22
Dudley..July 11-18, Aug. 8, 22, 29
Barrowden..June 20
East Dereham..August 29
Wisbeach..August 29
Pontesbury..August 8
Nottingham..June 13
Burton-on-Trent..June 20, July 11, [Aug. 22
Wrexham..August 1-8
Alford..July 4
Liverpool..June 13, July 4-11, Aug. 8, [22, 29
Warrington..August 1-8
Bolton-le-Moors..June 20-27, August 1, 8, 22

CHOLERA.

Chatham..July 11, August 1, 8, 22
Swansea..Aug. 1, 15 (Infantile ch.)
Newport Pagnell..August 1-8
Burton-on-Trent..August 29
Wrexham..Aug. 15-29 (English ch.)
Warrington..August 15
Bolton-le-Moors..June 27, July 18-25, [Aug. 8-22

AGUE.

Canterbury..June 13-27, Aug. 8-29
Chatham..Every week except July 25
Putney..August 1
Swansea..August 25
Bedford..June 6-20, July 11, Aug. 29
Sharnbrook..June 20-27, August 4
Newport Pagnell..June 27, July 18
Wellingborough..June 13 to July 18
Thetford..June 13, July 4
East Dereham..July 25, Aug. 1-8
Wisbeach..All June & July, Aug. 22-29
Alford..June 20, July 25 to Aug. 22
Gainsborough..July 18-25
Liverpool..August 22-29
Warrington..July 18

REMITTENT FEVER.

Teignmouth..June 6, 20, 27, July 11-[25, Aug. 15
Canterbury..July 11
Wandsworth..July 18, August 1
Putney..June 6, 13, 27, July 4, Aug. 8, [29
Upper Holloway..July 11-18
Wanstead..August 29
Swansea..July 18, August 8
Saffron Walden..June 6
Newport Pagnell..June 13
Beccles..June 6, 20, August 1-15
Thetford. July 11-18
Wisbeach..June 6-13, July 25 to Aug. [22
Burton-on-Trent..July 4, 18
Hawarden..August 8-22
Gainsborough..June 27, July 4
Liverpool..All August
Warrington..Aug. 8
Bolton-le-Moors..June 13, 27, July 4, [Aug. 1-8

DIARRHŒA.

Scilly Islands..June 6, 20, Aug. 8, 22, 29
Teignmouth..June 6-20, July 18 to [Aug. 29
Canterbury..Every week
Chatham..Every week
Wandsworth..All July and August
Putney..Every week except June 6
Upper Holloway..June 6-13, July 25 to [Aug. 29
Wanstead..Every week
Swansea..Every week [Aug.
Saffron Walden..June 6-13, July 11, all
Bedford..Every week except June 27
Sharnbrook..June 20 to August 29
Newport Pagnell..Every week except [June 13
Wellingborough..June 6-13
Beccles..July 11, August 15-29
Thetford..June 6, 20, 27, all July, Aug. 8, 15, 29
Dudley..Every week except June 6
Barrowden..June 27 to July 18
East Dereham..August 8-29
Wisbeach..June 6, 20, 27, July 18 to August 29
Pontesbury..July 18 to Aug. 29
Nottingham..August 1 to 29
Burton-on Trent..All June, July 25 to August 29
Wrexham..June 13-20, July 25 to Aug. [29
Hawarden..Every week except June 6
Alford..August 15-29
Gainsborough..June 27, July 4, 25 to [Aug. 29
Liverpool..Every week
Warrington..July 11 to August 29
Wigan..August 8-29
Bolton-le-Moors..June 27, July 4, 18 to August 22
York..June 13, 27, July 4-18, Aug. 8 29
Rothbury..July 11

DYSENTERY.

Chatham..June 13, August 22-29
Wandsworth..July 11, August 15
Upper Holloway..August 29
Wanstead..July 4
Swansea..July 11, 25
Newport Pagnell..June 20-27, July 4-[11, Aug. 1-8
East Dereham..Aug. 8-29
Wisbeach..August 22-29
Wrexham..June 27, July 4
Gainsborough..August 22-29
Liverpool..June 20, July 11-18, all Aug.
Bolton-le-Moors..July 4, 18, 25, Aug. [1-8
York..August 8, 22, 29

TYPHUS.

Teignmouth..August 15-22
Canterbury..Every week
Chatham. June 13, August 8-29

Wandsworth..June 6-20, July 18, Aug.
Putney..July 25, Aug. 1 [1, 8, 29
Bedford..July 18, August 8-29
Wellingborough..August 29
Thetford..June 6, 20, July 18, Aug. 22
Dudley..Every week except Aug. 29
East Dereham..August 22-29
Lincoln..August 1-22
Gainsborough..Every week
Liverpool..Every week
Wigan..August 8-29
Bolton-le-Moors..June 6-20, July 11, 25, August 1

PUERPERAL FEVER.

Beccles..July 11
Alford..June 27, August 1
West Auckland..June 6-20, Aug. 1 22

CARBUNCLE.

Teignmouth..June 20 [15-29
Canterbury..June 6-13, July 4, 18, Aug.

Chatham..August 1
Wandsworth..June 13, August 29
Wanstead..July 4
Saffron Walden..June 20, August 8
Sharnbrook..July 4
Newport Pagnell..June 20, Aug. 8-22
Beccles..July 4-18
Thetford..June 20, July 25
Barrowden..July 11
East Dereham..July 4-11
Burton-on-Trent..June 20
Wrexham..June 13
Liverpool..August 22-29
Wigan..July 4-18, August 1, 15
Bolton-le-Moors..July 11

MUMPS.

Canterbury..August 22
Wanstead..Every week

TYPHOID FEVER.

Swansea..August 8

---

### ADDITIONAL OBSERVATIONS.

*St. Mary's, Scilly.*—Mr. Moyle says: "Cases of catarrh, influenza, and pneumonia, were the most prevalent this quarter; latterly diarrhœa. I treated eighty cases of influenza in one week. There were no deaths in June, but seven occurred in July, in a population of 2,600; four from pneumonia, one from chronic bronchitis, one from disease of the mesenteric glands, and one (a child) from congestion of the brain. In August there were two deaths (an idiot and a child) from pneumonia. No disease has been prevalent among the cattle. We have had the usual amount of potato blight. The winds have been principally west, south-west, and north-west. The heat has been very great for Scilly in July and August, the temperature of 82° Fahr. being frequently attained; the mean summer temperature is 58°. There was scarcely any rain in June and July; in August there some heavy showers, the fall amounting in two days to 2·68 inches."

*Teignmouth.*—Mr. Lake writes: "The first eleven days of June were fine and pleasant, and were followed by a fortnight of colder and uncertain weather. From June 25th to July 9th there were a succession of warm and cool periods, each of a few days duration. From July 10th to 29th the weather was on the whole warm and close; the highest temperature for the season, 79·6°, occurred on the 19th. July 30th commenced a hot and brilliant week (five days being absolutely cloudless throughout), the barometric pressure varying in it from 30·258 to 29·970, the highest maximum temperature being 77·6, the mean maximum, 74·5, the mean minimum temperature 57·9, the mean humidity 68, mean ozone 1·6, the wind easterly. After three cooler days there followed another week of hot weather; in this the barometric pressure varied from 29.961 to 29·690, the highest maximum temperature was 79·1, the mean maximum temperature 75·7, the mean minimum temperature 60·4, the mean humidity 79, mean ozone 4·3; the wind chiefly from the S.W. and S., the sky clouded, and on two days showers of rain. After this the temperature fell somewhat considerably, and the weather was cooler and moister till the end of the month, the temperature rising again towards its close. The rain fall from May 30th to August 15th amounted only to 1·629 inch. Besides the epidemics mentioned in the reports, cases of chicken-pox continued to occur, and there were not a few of rheumatic fever, though not severe in character. During the hot weather roseola made its appearance, and there was a general prevalence of a sore and inflamed state of the gums and mouth. The diarrhœa of August and the latter end of July was generally accompanied with vomiting, and with a good deal of pain and soreness in the upper part of the belly, with some bilious suffusion of the skin, a furred tongue, and a tendency to a febrile condition, the motions being watery and dark-coloured."

Erratum. In the report for Teignmouth in the last number of the Journal of Public Health, p. 179, line 18 from the bottom, for "pleurisy", read "influenza".

*Canterbury.*—Mr. Rigden says: "During the past three months hooping-cough has continued to prevail in this city and neighbourhood. Diarrhœa has been prevalent during August. In the early part of June there were a few cases of scarlet fever, and a single case occurred in the third week in August. Only one case of measles and one of small-pox have come under my observation during this period. Fever of a typhous or typhoid character is seldom absent from this neighbourhood. Carbuncles continue to present themselves. The bills of mortality for this city show that deaths occurred in June from fever and croup; in July from fever, croup, hooping-cough, thrush, and diarrhœa; in August from fever and diarrhœa."

Mr. Reid furnishes the subjoined returns, drawn up by eight observers for the Epidemiological Committee of the East Kent and Canterbury Medical Society.

"*Abstract of Meteorological Observations for the Summer Quarter*, 1856.

| | *June.* Deg. | *July.* Deg. | *August.* Deg. |
|---|---|---|---|
| Highest temperature in the day time.... | 78 | 77 | 77 |
| Lowest.............................. | 50 | 52 | 55 |
| Mean ................................ | 62·36 | 63 | 65·322 |
| Highest temperature in the night ...... | 60 | 64 | 64 |
| Lowest.............................. | 45 | 42 | 48 |
| Mean ................................ | 52·26 | 54·019 | 57·12 |
| Highest reading of the barometer ...... | 30·18 | 30·14 | 30·10 |
| Lowest.............................. | 29·48 | 29·42 | 29·31 |
| Mean ................................ | 29·580 | 29·878 | 29·801 |
| Number of days on which rain fell...... | 11 | 14 | 13 |
| Amount of rain in inches.............. | 1·43 | 2·39 | 2·47* |

Direction of the wind (the number indicates the number of days the wind prevailed)..*June*—N. 3, NE. 5, SE. 1, S. 1, SW. 14, W. 1, NW. 5; *July*—N. 1, NE. 4, SE. 2, S. 2, SW. 13, W. 4, NW. 3 (only twenty-nine days observation); *August*—N. 3, NE. 4, E. 4, SE. 1, S. 2, SW. 13, W. 3, NW. 1.

"June. There were some sudden and considerable variations of weather observed during this month; the change from hot, close summer weather to a chilly temperature, produced by north-east wind, was much felt, and produced much general illness. Frosts were occasionally noticed at night. The days on which the greatest fall of rain occurred were the 1st, 14th, 16th, 19th, 20th, and 21st.

"July. Slight frost was observed on one or two nights this month; much storm rain fell during the month, and during the latter half there were several thunderstorms. The days of the greatest fall of rain were the 8th, 9th, 13th, 15th, 16th, 17th, 22nd, and 26th. On the 15th, between 12 A.M. and 2.45 P.M., 1·12 inch fell. From the fulness of the river, the valley became sodden during the heavy rains, and much damp generated in the lower part of the city.

"August. The heat during the latter part of July and the commencement of this month was very oppressive; the backward cereal crops were rapidly advanced by it. Frequent thunderstorms occurred; in the second week of the month there were four, accompanied by considerable fall of rain. The days of the greatest fall of rain were the 12th, 14th, 15th, 16th,* 17th, 19th, 20th, and 23rd. The rain much impeded the gathering of the harvest, and did some injury to the corn that was cut at the time.

"With two exceptions, the summer fruits were deficient,

* Two severe thunderstorms passed over the city during the night of the 16th, and a great body of rain fell, overflowing the rain-gauge; so that the rain could not be fully measured. The river was so swollen as to threaten a flood in the valley.

and there is a prospect of the autumnal fruits being scarce. The potato disease appeared rather earlier than last year, and was first noticed about the 18th.

*Abstract of the Returns of Zymotic Diseases during the Summer Quarter.*

"The number of cases, exclusive of diarrhœa, which have been returned by the eight observers during the summer quarter has been 70, being 96 less than the number reported during the spring quarter. This diminution, perhaps, is principally due to the subsidence of measles and small-pox, which chiefly prevailed in the early part of the year. The greatest number of cases was returned for the south-west or upper part of the valley district of the city; the next for the north-west or lower valley district; and the smallest for the east or higher district. The mortality in the whole number returned was at the rate of 10 per cent., and was occasioned by fever, hooping-cough, and croup.

"Small-pox has not yet left the city, and has supplied five cases during the quarter. In one instance it attacked a male, aged 33, who had three evident cicatrices of vaccination; it took a modified course. The four others who were attacked were members of one family who were unvaccinated; the disease presented the discrete form. The last case occurred in the beginning of August.

"Measles, which were shown to be gradually declining in each result of the spring quarter, appears to have ceased about the middle of July; only three cases have been returned during the period.

"Scarlatina is still present; eight cases have been noticed; six during June, and two during August. The cases all appear to have been mild. One child had anasarca of the ankles and face without albuminuria. No fatal case was reported.

"Continued fever shows a higher return this quarter; 19 cases have been returned; many cases of febricula were also noticed, but no report was made of them. The principal complications seem to have been head affection and diarrhœa. There were four fatal cases; three following head symptoms, and one diarrhœa.

"Ague. Seven cases of this disease were observed in June and August, presenting the three principal types; the tertian, however, predominated.

"Hooping-cough furnished eight cases, two of which were fatal from affection of the lungs. In one of those that recovered, croupy breathing was noticed for three days; and in another case there was slight pneumonia. The worst cases only come under observation, but the disease was reported as prevalent during June and July.

"Erysipelas. Eight cases of this disease were observed, being principally idiopathic, and of the head and face. No fatal case is mentioned.

"Mumps. An instance of this disease is noticed in August, but has not been observed before during the year.

"Diarrhœa, which was noticed in the last report as commencing in the latter part of the spring quarter, continued to be reported as prevailing during June and July, and during August a return of 53 of the severer cases was made by some of the observers. During the last month it has been the predominant epidemic, and is mentioned as such by all the observers. About 300 cases were treated at the hospital during August. One case, in a male of three years, is mentioned, as having fever with cerebral effusion supervening; a child of thirteen months had convulsions; another, four months old, six days after being attacked, had slight collapse; in another instance, it is mentioned that a twin sister had died in the same house without medical attendance. Beyond these, there is nothing to show that any of the cases had a choleraic type. It should be observed that fever and diarrhœa were noticed during every week of the quarter."

*Chatham.*—Dr. Brown writes: "In the middle of July two cases of serous cholera appeared. There were no more cases until the week ending 1st August, when there were a few mild cases. The following week there were some of the same description. On the 18th August there was an outburst of several severe cases. One house in the parish of St. Margaret's was particularly visited. A child of four years of age died, and the mother barely escaped with her life. The third case in the family was one of frequent vomiting, not amounting to cholera according to the ordinary meaning attached to the term. The child that died survived only twenty-one hours and a half. The weather on the 17th was unusual, there being a violent thunderstorm. The house that suffered so much was in a bad sanitary condition."

*Wandsworth.*—Mr. Nicholas writes: "Small-pox, which had prevailed to a great extent throughout the district since February, was gradually subsiding in July, when measles supervened; both diseases ceased at the beginning of August.

"During the months of July and August the prevalence of diarrhœa and of disease in general was much below the average; but in the week ending August 16th, a suddenly increased accession of that epidemic occurred. During that week there were no indications of the presence of ozone. During the last month the potato disease has been very destructive. Cattle have had entire immunity from epidemic disease."

*Putney.*—Mr. Whiteman states that "The quarter ending 29th August has been remarkably free from disease of the zymotic class. Diarrhœa of a mild form has prevailed more or less since the early part of June, and has increased within the last fortnight."

*Upper Holloway.*—The cases of remittent occurred in two children who had been resident a fortnight in the month of May at Erith, close upon Plumstead Marshes.

*Wanstead.*—Dr. Collins states that parotitis has prevailed throughout the quarter. Diarrhœa, though prevalent, is less severe and less general than usual.

*Saffron Walden.*—Mr. Stear says: "Scarlet fever has been the most important epidemic this quarter, and several fatal cases have occurred in the neighbourhood. Hooping-cough has also been prevalent, but the children have generally had it mildly. I have had several cases of diarrhœa during the past month. The harvest is being rapidly got up; the crops are good. The weather during the past quarter has been for the most part dry and hot; during the early part of August a good deal of rain fell."

*Beccles.*—Mr. Crowfoot writes: "The only prevalent epidemics with us during the last three months have been neuralgic affections and a severe form of diphtherite affecting primarily the mucous membrane of the pharynx, and in many instances extending to the larynx; the case of puerperal fever was an isolated one to which I was called in consultation, and which terminated fatally."

*Thetford.*—Mr. Bailey writes: "In this quarter, scarlatina, measles, and hooping-cough have been epidemic in the neighbouring villages, scarcely a family escaping the infection. Some cases of scarlatina have been very severe, assuming the type of scarlatina anginosa, requiring very active treatment, and great attention. In children dropsical effusions supervened, with swollen submaxillary glands. In our district no fatal case has been recorded. The temperature of June was exceedingly fluctuating; the mornings and evenings were cold, and the middle of the day very hot. The atmospheric pressure was variable. The prevailing winds were south-west, with only half an inch of rain during the month. This state of weather gave rise to several cases of catarrhal affections — influenza, and, with children, bronchitic disease, which terminated fatally in five cases. Amongst the labouring poor pleurisy was unusually prevalent, and also typhoid fever, of which one case proved fatal. Throughout the month erysipelas occurred in four instances. I cannot ascertain any communication with those affected with the disease. I consider it more epidemic than contagious. But few cases of diarrhœa occurred in June, and those only in the last week of the month, when the temperature rose to 90°—the highest during the summer in this locality. Rheumatism is a disease of both summer and winter, and may be attributed to imprudent exposure, to which the working classes often subject themselves. Several cases of cynanche tonsillaris came under treatment, arising from the same atmospheric cause.

"During July the atmospheric pressure was remarkably steady, except on the seventh and eighth days, and the temperature a summer one, the prevailing winds south-west, and the quantity of rain less than usual, being only 1·25 inch for the month. Diarrhœa was more prevalent, especially in children, which may probably be, in some measure, attributed to fruit; but with adults this was not the sole cause; the continued heat, with chilly evenings, more probably gave rise to the complaint. Only one case of typhoid fever came under medical observation in this month; the disease was cut short by large doses of quinine. Rheumatism and erysipelas still continued under treatment, and fresh cases were occurring. Influenza in an elderly person proved fatal. Two cases of remittent fever occurred in labourers, arising from being wet many hours during their work; in one of whom a large carbuncle formed in the neck. The casual diseases under notice were paralysis, gastralgia, menorrhagia, gout, and some cutaneous diseases.

"For several years we have not experienced so hot a summer as in part of July and the first fortnight of August, the temperature averaging 78°. The prevailing winds were north-east half the month, and south-west the other half. The rain amounted to only 0·75 inch. Scarlatina, measles, and hooping-cough still prevail around us in August, attacking adults as well as children. In several of the latter dropsical effusions, with swollen glands occurred, being produced by too early exposure. However, there was no fatal case in the whole district. On the 18th of August a considerable reduction of temperature took place, and continued to the end, averaging as low as 63°. This vicissitude gave rise to many cases of bronchitic affections among children, and influenza and catarrhal affections in adults, also to some severe attacks of asthma in those predisposed to the disease. Erysipelas was still epidemic, attacking the face, head, and the epigastric region, producing much constitutional derangement. Diarrhœa was not so prevalent as in July; but rheumatism was more frequent with the working classes. During the excessive heat the harvest men were suddenly attacked with violent spasmodic pains and cramps in the stomach and bowels, evidently arising from drinking cold water or small beer while in a state of great perspiration. Large doses of opium with aromatics speedily relieved them; but the prostration left prevented them from resuming their work for some days. The casual diseases of the month were leucorrhœa, hysteria, hepatitis, laryngeal consumption, abortion, placenta prævia, fracture, hæmorrhoids, hernia humoralis, gonorrhœa, headache, tic douloureux, and neuroses.

"Pneumonia and enteritis have been the prevailing diseases among the cattle, and also the strangles. From the abundance of food, the sheep are more healthy, but great mortality previously existed from the murrain. There has been a great failure of fruit, especially apples; and the leaves indicate an early autumn."

*Record of Deaths in Twenty-one Parishes (population 9,574) from the Registrar's Book.*

| *June.* | | *July.* | | *August.* | |
|---|---|---|---|---|---|
| Diseased heart .... | 2 | Bronchitis ........ | 3 | Paralysis .......... | 1 |
| Atrophy ........... | 3 | Atrophy ........... | 1 | Hooping cough .... | 1 |
| Inflammation of brain | 1 | Debility .......... | 2 | Decay of nature .... | 1 |
| Consumption ...... | 6 | Cephalitis ........ | 1 | Consumption ...... | 3 |
| Premature birth.... | 1 | Decay of nature .... | 2 | Mesenteric disease.. | 1 |
| Debility .......... | 2 | Diseased heart .... | 1 | Diseased heart .... | 1 |
| Convulsions ...... | 1 | Consumption ...... | 3 | Hydrocephalus .... | 1 |
| Drowned .......... | 1 | Hooping cough .... | 1 | Congestion of brain | 1 |
| Natural decay...... | 1 | Dropsy............ | 1 | | |
| Fever ............ | 1 | | | | |
| | 19 | | 15 | | 10 |

*Dudley.*—Mr. Houghton writes: "Dudley has been remarkably free from zymotic diseases during the last thirteen weeks. Cases of fever and diarrhœa have occurred in each week of the quarter, but they have not been numerous or severe. Inflammations, of a phlegmonous character, have been prevalent, chiefly attacking the hand and fingers, and requiring free and early incisions. The cases of erysipelas have assumed the same type. In two cases it was associated with albuminous urine, with fatty casts in the one case, and granular casts in the other. The former was fatal; the latter recovered partially, after having had convulsions and paralysis. Pleuropneumonia has attacked horses, and influenza cattle generally; glanders have been prevalent."

*Barrowden.*—Mr. Swann states that, in the Barrowden district, consisting of thirteen parishes, diarrhœa was very prevalent during the latter part of June and beginning of July, for two or three weeks. During the last five weeks measles had been spreading rapidly through North Luffenham; but no deaths had occurred.

*Burton-on-Trent.*—Dr. Thomson says: "Diarrhœa was more prevalent than usual about the middle of June. On the 28th of June a case of remittent fever, traceable, I believe, to a few days residence close to where the cleanings of large pool had been placed. The weather was at the time extremely hot. On August 23rd I was called about 9 P.M. to a man, aged 30, who had been seized with choleraic symptoms. During the day, from 9 A.M., rice water purging had been going on to a large extent, with frequent vomiting; at 3 P.M. cramps came on in the extremities. When he was visited, the purging and cramps still continued, the surface was cold and blue, the extremities of the fingers shrivelled, the tongue cold, the voice suppressed, the pulse scarcely perceptible; the whole appearance being one of intense collapse. The urine was completely suppressed for thirty-six hours. Under proper treatment the man recovered.

"This was a case of aggravated British cholera, which it would be extremely difficult to distinguish from a somewhat severe form of the Asiatic disease. In the case in question, there was very copious rice water or gruel purging going on for some hours, accompanied with sickness; in a few hours cramps set in, the surface became cold and blue, the fingers shrivelled, the tongue cold, the voice much reduced in power and altered in pitch, pulse almost imperceptible, thirst intense: and there was suppression of urine for thirty-six hours. There was no sweating and no consecutive fever; the disease at once yielding to a mixture composed of chloroform and liquor opii in strong doses, given in conjunction with pills of gallic acid, and aided by a very efficient vapour bath.*

* I take this opportunity of noticing the "very efficient vapour-bath", which I am now constantly in the habit of using, both on account of its efficiency,

"The village in which this case occurred is very badly drained, a large open ditch or sewer running close to the man's house. Moreover, for some days previously, a more than usual amount of bilious cholera had prevailed among his neighbours, and his own child had been severely affected. It appears to me that the absence of consecutive fever strongly marks the difference between the more specific simple British cholera, that is, a non-malignant disease, commencing, or at least distinctly marking its commencement in the chylopoietic viscera, and the specific malignant Asiatic disease commencing, probably, in the nervous system, and precipitating, as it were, in most instances, but not in all, its influence upon the alimentary tract. The one simple disease, the other as essentially specific as either typhus or typhoid fever."

*Lincoln.*—Mr. Lowe writes: "This district has been more than usually free from epidemic and endemic diseases during the past quarter. A few cases of typhus fever, occurring in some newly built houses, in a low situation, are all I have had to note."

*Stavely.*—Mr. Thorpe says: "There has not been an epidemic of any description the last quarter; in fact, the neighbourhood generally is in a much healthier condition than I have known it for the sixteen years I have resided here. This I attribute, in a great measure, to the attention paid to draining and sewerage."

*Gainsborough.*—Dr. Mackinder writes: "Notwithstanding the great fluctuations in the weather, Gainsborough has been exceedingly healthy during the past quarter. Diarrhœa and English cholera have been much less frequent than usual at this season, owing, perhaps, to the great scarcity of fruit. In August, as observed by Mr. Dyson,

---

and of its convenience, especially in the houses of the poor, where really good means of applying general heat are so completely wanting. The method may be familiar to some; but to others it may be, as it was to myself, a welcome addition to my ways and means. Three or four new porous bricks are to be boiled in water for an hour, or longer if there is no hurry; they are to be taken out steaming hot and laid on the floor; the patient is seated on a chair placed over the bricks, and a blanket, thrown over all, is to be fastened round his neck. In a very few minutes the person is perfectly enveloped in the steam, which is kept up by the saturated and heated bricks as long as it is required, and all the effects of the vapour-bath most perfectly attained. It will be found a most valuable mode of restoring heat. In the case above cited, the patient said he felt better "ever after getting warm over the bricks". For this useful application I am indebted to Mr. Twining, surgeon, of Appleby, in this neighbourhood. S. T.

the amount of ozone was excessive; but whether this has had anything to do with our salubrity or not I cannot decide. I have had one case of tertian ague, imported at the middle of July, when there was a little troubleseme brow ague about. In a village four miles north of us typhoid has been endemic throughout the quarter. The portion of the village in which the fever has located itself is considerably elevated; but the houses are small, ill-ventilated, and exposed to noxious effluvia from pigsties and gutters. An old woman died of typhoid fever; her grandchild took the disease and recovered. Its mother, who afterwards came from a town sixteen miles off, in a state of health, was prostrate in a fortnight from the same affection. The village is a rather large one, but poor, and the fever has hitherto been confined to a few houses at one extremity."

The subjoined meteorological report has been furnished by Mr. Dyson: "The meteorology of the quarter ending August 31st was characterised by the extreme heat of the early part of August, the maximum being 93°, and the unusual amount of rain which fell during the same month, the amount collected having been 3·7, or nearly 4 inches. The month of June was cold and showery till the 25th; July hot and fine, especially towards the close. An unusually large amount of ozone was also developed during the month of August, during which frequent and heavy showers of rain fell. The greatest heat of June was 75° on the 10th, the lowest 41° on the 14th. The hottest day in July was the 31st—90°, and the coldest night the 24th—48°. In August the hottest day was the 1st—97°, and the coldest night, 45°, on the 29th. The contrasts during the quarter were the intense heat of July and August, and the wetness of the latter month."

*Liverpool.*—Mr. Bickerton makes the following report:—"June 8th, 1856. During the past seven days we have been favoured with exceedingly fine weather. Some days have been very hot, but rain has fallen during the night or early morning. There has been a great diminution in the amount of sickness and the number of deaths, viz., 176, is far lower than in corresponding week during last eight years; the corrected average for the same week being 223. Scarlatina caused 4 deaths; measles, 5; small-pox, 1 (primary vaccination); typhus, 9; hooping-cough, 8; syphilis, 1; diarrhœa, 5; diseases of chest, 58; drunkenness, 3: in females, 1, aged 38, ending in apoplexy; 1, aged 38, delirium tremens; and 1, aged 40, from suffocation.

" June 21. During the week 186 deaths have been registered. Scarlatina caused 7 deaths; measles, 10; small-pox, 3 (one not having been previously vaccinated); typhus, 4; hooping-cough, 13; diarrhœa, 7; syphilis, 3; diseases of lungs 64 (32 being from consumption).

" During the quarter ending 21st June, 2,628 deaths were registered, including 159 sudden and violent deaths: being 517 less than in the preceding quarter, and about 600 less than the corrected average for same quarter of last two years. The deaths from zymotic diseases were 535, being 223 less than the average. Scarlatina caused 90 deaths; measles, 90; small-pox, 18; diarrhœa, 60; hooping-cough, 100; typhus, 80; quinsy, 36; cholera, 2; erysipelas, 17; syphilis, 23; diseases of lungs, 850.

" July 5th. During this week 161 deaths have occurred, being a lower mortality than has been registered in any week of the last five years, and 61 below the average of the preceding eight years, for the corresponding week. Scarlatina caused 6 deaths; measles, 16; small-pox, 2 (neither having been vaccinated); diarrhœa, 8; typhus, 7; hooping-cough, 3; diseases of lungs, 49 (32 from phthisis); delirium tremens, 1. The mean temperature was 59·8. The atmosphere has been dry, and reading of barometer has been high. No rain fell during the week excepting a very slight shower on Monday.

" July 12. There have been during the week 176 deaths. Scarlatina caused 12 deaths; diarrhœa, 11; hooping-cough, 8; measles, 6; typhus, 5; small-pox, 2; syphilis, 2; purpura, 1; diseases of chest (including consumption), 45

" July 19. The number of deaths this week has been 175, being 41 less than the average. Scarlatina caused 6 deaths; measles, 9; small-pox, 1; diarrhœa, 17; typhus, 9; syphilis, 1; mumps, 1; diseases of lungs, 49; intemperance, 2.

" July 26th. During the past week there have been 179 deaths, the average being 256. Zymotic diseases caused 53 deaths; scarlatina, 6; measles, 6; small-pox, 3; common continued fevers, 4; typhus, 1; diarrhœa, 29; diseases of the brain and convulsions, 33; diseases of the liver, 2; dropsy, 3; diseases of lungs, 39; and diseases of the heart, 6.

" August 3. In this week 201 deaths have been registered, being 71 below the average. Scarlatina caused 9 deaths; measles, 8; small-pox, 1; diarrhœa, 30; typhus, 2; hooping-cough, 4; quinsy, 2; simple fever, 6; diseases of brain, 34; diseases of lungs, 37; old age, 28; diseases of heart, 5; painter's colic, 1; dropsy, 4. The thermometer on Saturday was 84° in the shade, being about four degrees higher than any day during the last ten years. The mean reading of the barometer for the week was 30·17 inches.

"August 10. There have been 234 deaths during past week, being 68 below the corrected average. The temperature of the week has been very high, Saturday, August 4th, being the highest, when the thermometer stood at 85·09° in the shade. Scarlatina caused 7 deaths; measles, 5; small-pox, 2; fever, 6; hooping-cough, 6; laryngitis, 2; diseases of brain, 44; diarrhœa, 66 (55 being children under two years old); diseases of lungs, 45; debility and old age, 26; diseases of liver, 4; tetanus, 1.

"August 17th. There were 226 deaths registered this week, being 76 fewer than the corresponding average for the same week in previous eight years. Scarlatina caused 4 deaths; measles, 4; diarrhœa and diseases of bowels, 67; hooping-cough, 4; fever, 2; small-pox, 1; laryngitis, 3; diseases of brain, 18; convulsions, 17; diseases of lungs, 36; old age, 33; diseases of heart, 5; liver, 1; dropsy, 3. There has been a great fall in the temperature from the previous week.

"August 24th. There have been 252 deaths registered during the past week, being 32 less than the average. The great heat has caused an increase in the deaths from diarrhœa. Scarlatina caused 15 deaths; measles, 4; small-pox, 4; hooping-cough, 5; diarrhœa, 75; diseases of the lungs, 34; diseases of brain, 36; old age, 19; diseases of the heart, 4; delirium tremens, 1. The mean temperature has been 65·5°; the mean barometric pressure 29·71 inches.

"August 31. During the week 230 deaths have been registered, being 56 less than the average of preceding eight years. Scarlatina caused 13 deaths; measles, 3; small-pox, 2; diarrhœa, 69 (63 being children under two years of age); typhus, 2; hooping-cough, 3; diseases of brain, 27; diseases of lungs, 45; old age, 10; destitution, 6;* epilepsy, 2; cancer, 4; dropsy, 2; delirium tremens, 1.

"From inquiries made, I cannot find that either animals or plants have suffered from any epidemic diseases during the past three months; on the contrary, there has been generally a remarkable freedom from any serious epidemic affections in this neighbourhood."

*Wigan.*—Mr. Cox writes: "The most prevalent epidemics

* I cannot understand how this can occur in Liverpool, where the parochial matters are so regularly managed. It certainly looks bad. T. B.

during this quarter have been hooping-cough and croup, occurring as the sequelæ of measles in most cases. They have also shown a very high ratio of mortality. Of seven cases of croup, three proved rapidly fatal. Of thirty-two cases of hooping-cough, five were fatal, by the supervention of extensive lobular pneumonia. The outbreak of measles, which had been terribly severe and of the malignant type, during the last quarter, declined and disappeared during June. The recent cases of diarrhœa have been severe, some of them choleraic; but none that came under my own notice proved fatal. The wind during June and July was chiefly south-east and east, with a very low temperature; during August it was west and north-west, with a high temperature. There was an unusual amount of rain and lightning."

*York.*—Mr. Procter says: "During the quarter the health of the city has been better than is usual at this period of the year. There has been much less diarrhœa, that though in my cases assuming a dysenteric character, has readily submitted to medical treatment. Several cases of small-pox, mild in their character, have shown themselves."

*West Auckland.*—Mr. Todd says: "The cases of small-pox were all after vaccination, and were modified and of a slight nature. The cases of puerperal fever were of a low typhoid nature, of that form called puerperal phlebitis. One such case proved fatal, and was attended with secondary deposits, the lungs becoming much affected.

*Rothbury.*—Mr. Summers writes:—"This quarter has been marked by an almost total absence of epidemic disease in this neighbourhood. Congestive diseases, affecting chiefly the lungs and liver, have been prevalent, as also has rheumatism. There still prevails the asthenic tendency which I noticed in my last report. With the exception of a few days in August, the season has been in this district very cold and backward."

